# Online Antenatal Care During the COVID-19 Pandemic: Opportunities and Challenges

**DOI:** 10.2196/19916

**Published:** 2020-07-22

**Authors:** Huailiang Wu, Weiwei Sun, Xinyu Huang, Shinning Yu, Hao Wang, Xiaoyu Bi, Jie Sheng, Sihan Chen, Babatunde Akinwunmi, Casper J P Zhang, Wai-Kit Ming

**Affiliations:** 1 Department of Public Health and Preventive Medicine School of Medicine Jinan University Guangzhou China; 2 Faculty of Medicine, International School Jinan University Guangzhou China; 3 Department of Culture and Tourism Xiamen University of Technology Xiamen China; 4 College of Economics Jinan University Guangzhou China; 5 Maternal-Fetal Medicine Unit, Department of Obstetrics and Gynecology Brigham and Women’s Hospital Boston, MA United States; 6 Center for Genomic Medicine, Massachusetts General Hospital, Harvard Medical School Harvard University Boston, MA United States; 7 School of Public Health, Li Ka Shing Faculty of Medicine The University of Hong Kong Hong Kong China

**Keywords:** coronavirus disease, COVID-19, SARS-CoV-2, online prenatal education, pregnancy, online education, antenatal, telehealth, convenience, inequality

## Abstract

People across the world have been greatly affected by the ongoing coronavirus disease (COVID-19) pandemic. The high infection risk of severe acute respiratory syndrome coronavirus 2 (SARS-CoV-2) in hospitals is particularly problematic for recently delivered mothers and currently pregnant women who require professional antenatal care. Online antenatal care would be a preferable alternative for these women since it can provide pregnancy-related information and remote clinic consultations. In addition, online antenatal care may help to provide relatively economical medical services and diminish health care inequality due to its convenience and cost-effectiveness, especially in developing countries or regions. However, some pregnant women will doubt the reliability of such online information. Therefore, it is important to ensure the quality and safety of online services and establish a stable, mutual trust between the pregnant women, the obstetric care providers and the technology vis-a-vis the online programs. Here, we report how the COVID-19 pandemic brings not only opportunities for the development and popularization of online antenatal care programs but also challenges.

## Introduction

The coronavirus disease (COVID-19) outbreak has spread globally and caused a pandemic that has led to almost 10,000,000 diagnosed cases and 500,000 deaths as of June 28, 2020 [1]. The impact of COVID-19 can be greater in vulnerable populations. Pregnant women, for example, tend to be more physiologically and psychologically susceptible to infectious diseases, putting them at higher risk of maternal complications such as preterm birth, gestational hypertension, gestational diabetes, and miscarriage [2-4]. Appropriate antenatal education can be beneficial to them in many ways including reducing cesarean section rates, maternal and infant mortality, and anxiety problems as well as improve their general reproductive health outcomes [5]. In the context of the pandemic, pregnant women face an additional dilemma—they need professional antenatal care, there is a potential risk for cross-infection if they choose to visit a hospital to receive this service [6].

**Figure 1:**
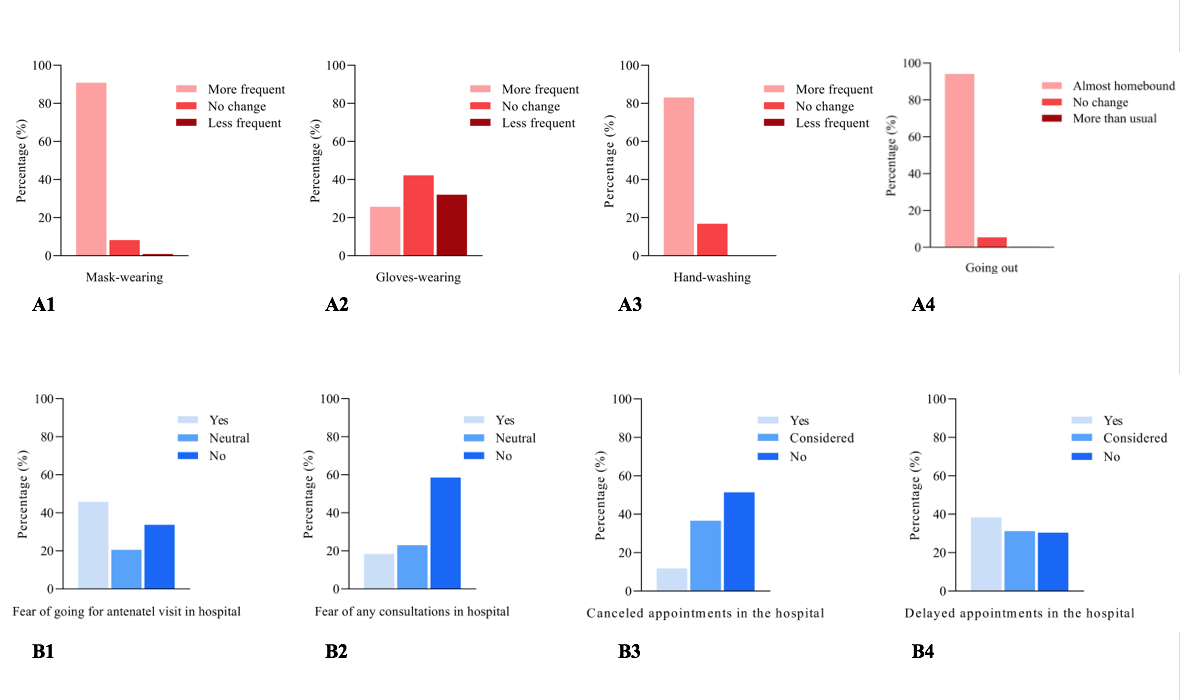
Pregnant women’s self-protection behaviors and attitudes toward antenatal care in hospitals.

Recently, we performed a web-based survey among Chinese pregnant women via a national online platform (Banmi National Online Maternity School) to investigate their self-protection behaviors and attitudes toward antenatal care during the pandemic. A total of 983 Chinese pregnant women completed the questionnaire, and it was found that more than 80% had taken self-protection actions, such as wearing a face mask, handwashing, and home quarantine, to avoid being infected with severe acute respiratory syndrome coronavirus 2 (SARS-CoV-2) (Figure 1). Our findings indicate that about 20% of respondents were afraid of any type of consultation at a hospital, while over 40% feared in-hospital antenatal visits. Moreover, more than half considered or decided to cancel their in-hospital antenatal care visits and postponed their appointments. These behaviors and attitudes indicate that pregnant women were anxious and worried about potential infection in the hospital environment. Considering the dilemma mentioned above and the fear of some other unknowns from hospital visits, online antenatal care might be a preferable choice for pregnant women during this pandemic [7].

## Online Antenatal Care Programs Before the COVID-19 Pandemic

Online antenatal care programs can take various forms: online courses to deliver pregnancy-related information, mobile phone apps to motivate healthy behaviors during pregnancy, and mobile health (mHealth) apps to provide mental consultations [8,9]. Numerous studies have reported that a great majority of pregnant women search for pregnancy-related information on the internet [10,11]. However, the popularity of online antenatal care programs remains low.

## Opportunities for Online Antenatal Care During the Pandemic

In the midst of the COVID-19 pandemic, it is advisable that pregnant women should stay at home and receive necessary antenatal care via online antenatal care programs. For example, online antenatal care programs may guide pregnant women to perform blood and urine glucose tests at regular intervals, especially for those with or at high risk of gestational diabetes mellitus (GDM). The obstetric doctor can then closely monitor patients’ glucose levels and provide appropriate dietary suggestions and medications. This would be helpful in decreasing the incidence and negative impact of GDM. Moreover, women can upload their daily blood pressure and simple home urine dipsticks results to an online system. This would allow monitoring for serious pregnancy complications such as preeclampsia. General gestational education and mental health consultation can be done through an online education program via voice or video calls. Pregnant women can also be guided to study pregnancy and labor instructions through books, and receive antenatal care education by online conferencing. All of these approaches would likely contribute to better labor preparedness, maternal experience, postpartum adjustment and outcomes, fewer infection risks, and more economic benefits to the health system and the women themselves. Therefore, pregnant women without any serious issues should use online antenatal care programs as an alternative to routine antenatal care in a hospital at least to some extent. Learning to self-monitor while in lockdown or home quarantine is essential to prevent viral infection or spread [12]; this is especially important to pregnant women as they are more likely to have severe complications if infected with SARS-CoV-2 [13-15].

During the pandemic, daily necessities were scarce and the cost of medical appointments was substantially higher than usual. Many families lost employment and suffered a heavy economic burden. People who were unable to obtain or pay for sufficient medical resources were most vulnerable [16]. Online antenatal care can provide relatively less expensive medical services and diminish inequalities in health care due to its convenience and cost-effectiveness, especially in developing countries or regions. This could contribute to a reduction in medical resources inequality and help pregnant women resolve various health problems during the pandemic. Medical experts and institutions should strengthen the quality of online antenatal care in terms of skilled professional services, technology, and availability.

## Challenges of Online Antenatal Care During the Pandemic

There are some challenges and areas for improvement associated with online antenatal care. A study in China found that many pregnant women had concerns about the reliability of online gestational information [17]. Therefore, it is important to establish close collaboration between hospitals and professional institutes to improve the quality of online programs, ensuring the reliability of their information. In the meantime, this can be combined with information technology products, such as using electronic devices with remote monitoring functions to monitor basic indicators such as fetal heart rate and movement, to serve as a proxy for some routine obstetric examinations. However, in certain circumstances, antenatal care in hospitals is irreplaceable (eg, high-risk pregnant women with or at risk of vaginal bleeding, abdominal pain, or other serious discomforts) [18]. In such cases, further obstetric examinations and consultations in a hospital are essential. Moreover, some specific antenatal examinations such as the Nuchal Translucency test, Down syndrome screening, and the Oral Glucose Tolerance Test should be completed in a hospital. The future of maternal care is likely to include technological innovations to address the above challenges. Due to this, more extensive, optimized maternal care services should be applied to reduce overall maternal morbidity and mortality if online antenatal care are to be further developed, popularized, and adopted as an alternative path to health care services for pregnant women.

Furthermore, it has been mentioned that less than one-third of the population in Africa and the Middle East use the internet, with a global usage rate of 51% in 2018 [19]. Therefore, in addition to improving online antenatal care, the popularization of both the use of the internet and mobile electronic devices is crucial to allow more pregnant women to receive online education and care. Network operators need to be improved to guarantee the widespread use of internet services during the COVID-19 outbreak. Governments should use their financial budget to support the popularization of modern electronic devices and internet service, which are essential for online antenatal care programs. Additionally, governments need to legislate relevant laws to regulate and protect the privacy of pregnant women when using online antenatal care services.

## Conclusion

In summary, online antenatal care can be a useful, alternative option for pregnant women in need of basic antenatal care and mental health consultation. It can reduce unnecessary hospital visits and limit potential risks of infection among this vulnerable group during the COVID-19 pandemic. Efforts to implement online care is likely to result in multiple innovations and revolutionize antenatal care services both in China and globally. This will contribute to reducing maternal morbidity and mortality by providing opportunities for wider coverage. The popularization of online antenatal care programs is likely to have an economic benefit to both the health care system and to women in terms of cost, time, and manpower. This can improve overall maternal and reproductive health services and family life.

## Abbreviations

COVID-19: coronavirus disease
GDM: gestational diabetes mellitus
mHealth: mobile health
SARS-CoV-2: severe acute respiratory syndrome coronavirus 2
